# Identification of a two metastasis-related prognostic signature in the process of predicting the survival of laryngeal squamous cell carcinoma

**DOI:** 10.1038/s41598-023-40740-2

**Published:** 2023-08-19

**Authors:** Yuebin Zheng, Jun Wu, Bincheng Yan, Yirong Yang, Huacai Zhong, Wang Yi, Chengjian Cao, Qian Wang

**Affiliations:** Department of Otolaryngology Head and Neck Surgery, Zigong First People’s Hospital, Zigong, Sichuan China

**Keywords:** Cancer genetics, Cancer genomics, Cancer therapy, Head and neck cancer

## Abstract

Metastasis is a major cause of treatment failure and poor outcomes in cancer patients. The data used in the current study was downloaded from TCGA and GEO databases. Differentially expressed metastasis-related genes were identified and the biological functions were implemented. Kaplan–Meier analysis univariate, and, multivariate Cox regression analyses were performed to identify robust prognostic biomarkers, followed by construction of the risk model and nomogram. Gene set enrichment analysis was performed to identify pathways enriched in low- and high-risk groups. *POLR2J3* and *MYH11* were treated as prognostic biomarkers in LSCC and the risk model was constructed. Receiver operating characteristic curves revealed the good performance of the risk model. A nomogram with high accuracy was constructed, as evidenced by calibration and decision curves. Moreover, we found that the expressions of *POLR2J3* and *MYH11* was significantly higher in metastasis tissues compared with those in non-metastasis tissues by RT-qPCR and IHC. Our study identified novel metastasis-related prognostic biomarkers in LSCC and constructed a unique nomogram for predicting the prognosis of LSCC patients. Moreover, we explored the related mechanisms of metastasis-related genes in regulating LSCC.

## Introduction

In recent years, laryngeal cancer has become one of the most common human head and neck tumors, accounting for one-third of all head and neck cancers, and is the sixth most common cancer in the world^1^. However, according to a large case series study published by Ciolofan et al.^2^, laryngeal squamous cell carcinoma (LSCC) accounted for more than 98% of the histological types of laryngeal cancer. In recent years, despite LSCC can be treated by a combination of surgery, radiotherapy, chemotherapy and biological therapy, the survival rate of laryngeal cancer is still very low regardless of the treatment plan, and the 5-year overall survival rate is 50–70%^3^. Metastasis is one of the main reasons leading to treatment failure and poor prognosis of cancer patients^4^. At present, the clinical TNM staging system is used to evaluate the prognosis of LSCC, however, the prediction effect is not satisfactory^5^. Current studies have found that there are different genes abnormally expressed in the process of occurrence, invasion, and metastasis of laryngeal cancer, such as *FBXL20*^6^, *PRR4*^7^, *HMGA2*^8^, *miR-552*^9^, etc. However, the specific mechanism of action is unknown, and the specificity of early diagnosis of LSCC is not clear. The effect of predicting the survival rate of LSCC patients remains to be explored. Therefore, identifying genetic signatures and potential biomarkers of LSCC aggressiveness will help predict the survival or recurrence odds of the disease. In addition, the development of potential important biomarkers will make it possible to achieve precise treatment of individual cases, and provide a certain experimental and theoretical basis for further targeted therapy.

Since biomolecules interact to function in biological processes in cells and tissues, comprehensive analysis in the context of network medicine is essential to understand the molecular mechanisms behind diseases and identify key biomolecules. In this study, we screened differentially expressed metastasis-associated genes in TCGA and GEO databases by bioinformatics analysis and identified, *POLR2J3* and *MYH11* as prognostic biomarkers of LSCC by univariate and multivariate Cox regression analysis. The quantitative real-time polymerase chain reaction (qRT-PCR) was performed in laryngeal cancer tissues and adjacent tissues. The qRT-PCR and the immunohistochemistry (IHC) were used to verify the expression of two prognostic biomarkers, *POLR2J3* and *MYH11*.

## Materials and methods

### Ethics statement

From September 2020 to September 2021, 20 patients with laryngeal squamous cell carcinoma who underwent surgery in the Department of Otorhinolaryngology Head and Neck Surgery, the First People’s Hospital of Zigong were collected, including 10 cases of non-metastatic laryngeal squamous cell carcinoma samples and 10 cases of laryngeal squamous cell carcinoma samples with metastasis. All patients underwent the first operation. The preoperative diagnosis was laryngeal carcinoma, pathological type was squamous cell carcinoma, and no preoperative chemoradiotherapy was performed. The study has been approved by the Ethics Committee of the First People’s Hospital of Zigong City and informed consent of patients, Ethics number: Ethics (Research) No. 8, 2021.

### Data source

Gene expression profiles and clinical information of laryngeal squamous cell carcinoma (LSCC) patients were downloaded from TCGA (https://www.genome.gov/Funded-Programs-Projects/Cancer-Genome-Atlas) and GEO (https://www.ncbi.nlm.nih.gov/geo/) databases. Specifically, we extracted 80 LSCC patients with complete clinical information from TCGA-HNSCC cohort and used them as the training set. In addition, 56 LSCC patients with survival information in GSE25727 were used as the external validation set.

### Identification and functional analysis of DEMRGs in LSCC

DESeq2 package^10^ was used to screen differentially expressed genes (DEGs) between 38 samples at N0 stage and 42 samples at N1/N2/N3 stage using |log_2_FC| > 1 and *p* value < 0.05 as criteria. Those DEGs were defined as differentially expressed metastasis-related genes (DEMRGs). Gene ontology (GO), including biological process (BP), cellular component (CC) and molecular function (MF), and Kyoto Encyclopedia of Genes and Genomes (KEGG) enrichment analyses of DEMRGs were performed by clusterProfiler^11^ in R package. A *p* value < 0.05 was considered as significantly enriched.

### Identification of prognostic DEMRGs

After removing DEMRGs with that were not expressed in more than half of the samples, the remaining DEMRGs were input into univariate Cox regression analysis to select DEMRGs associated with survival (*p* value < 0.05). Meanwhile, LASSO regression analysis was performed by glmnet R package to further screen gene signatures at Lambda.min. Then according to the expression of each gene signature, LSCC patients were divided into low- and high-expression groups. Disease-free survival (DFS) survival analysis and log-rank test were used to analyze and compare the survival between low- and high-expression groups, respectively. Thereafter, gene signatures with significant survival difference between low- and high-expression groups (*p* value < 0.05) were input into multivariate Cox regression analysis to get more robust prognostic DEMRGs (*p* value < 0.05).

### Construction of the risk model and nomogram

The risk score was calculated as the following formula:

$$ {\text{ExpGene1}}*{\text{Coef1}} + {\text{ExpGene2}}*{\text{Coef2}} + {\text{ExpGene3}}*{\text{Coef3}}^{12} $$

where Coef means the regression coefficients of prognostic DEMRGs, Exp is the normalized expression values of each prognostic DEMRGs. According to the median value of the risk score, LSCC patients in the training set were divided into low- and high-risk groups. DFS curves were plotted to investigate the survival difference of LSCC patients in low- and high-risk groups. ROC curves for 1-, 3-, 5- and 7-year were plotted using the “survivalROC”^13^ in R package, and the larger the AUC value, the more accurate the risk model was in predicting survival time of patients. The above analyses were performed in GSE25727 dataset to validate the risk model. Thereafter, univariate and multivariate Cox regression analyses were carried out to determine independent prognostic factors for LSCC patients (*p* value < 0.05). Based on independent prognostic factors, a nomogram was established to predict 1-, 3- and 5-year survival of LSCC patients. We scored the sample for each factor (including N, Stage and risk score) and calculated the total point, with higher scores associated with lower survival rates.The performance of the nomogram was evaluated by the calibration and decision curves.

### Construction of PPI network in LSCC

The KEGG and GSEA analyses were performed to explore the function of high-and low-risk groups. GSEA is used to evaluate the distribution trend of predefined genes in a gene list ranked by their phenotypic correlation, and thus determine their contribution to the phenotype. However, KEGG analysis required first screening DEGs between high and low risk groups by DESeq2 package using |log_2_FC| > 1 and *p* value <0.05 as criteria. The KEGG pathway enrichment of DEGs was analyzed by clusterProfiler R package. A *p* value < 0.05 was considered as significantly enriched. Then DEGs were uploaded to STRING^14^ (https://string-db.org/) database to explore their interactions. Cytoscape software was used to construct and visualize the protein-protein interaction (PPI) network of DEGs.

### RT-qPCR

Total RNA of metastatic LSCC samples (n = 10) and non-metastatic LSCC samples (n = 10) were extracted by Nuclezol LS RNA Isolation Reagent (ABP Biosciences Inc, China). After detecting the concentration and the purity of RNA, qualified RNA was used for reverse transcription using SureScript-First-strand-cDNA-synthesis-kit (GeneCopoeia, USA). Then qPCR on a CFX96 Real-time PCR System (Bio-Rad, USA) was performed using BlazeTaq™ SYBR ^®^ Green qPCR Mix 2.0 (GeneCopoeia, USA) under the thermal cycling conditions: 40 cycles at 95 °C for 30 s, 95 °C for 10 s, 60 °C for 20 s and 72 °C for 30 s. The 2^−∆∆Ct^ method was used to calculate gene expressions. The primer sequences used in the current study were given in Table 1.

**Table 1:** Primers used in the current study.

Genes	Forward	Reverse
*POLR2J3*	CAGCCTTCGAGTCGTTCTTG	AATAGCACTTGCGGGTCTTT
*MYH11*	AGCGGCAACTCGTGTCCAAC	CTCCTCATTCTGCTCGTCCC
*GAPDH*	CGCTGAGTACGTCGTGGAGTC	GCTGATGATCTTGAGGCTGTTGTC

### IHC analysis

Paraffin embedded metastatic LSCC samples (n = 10) and non-metastatic LSCC samples (n = 10) were sectioned, deparaffinized, and rehydrated. For antigen retrieval, sections were treated for 10 min with antigen retrieval reagent (Biosharp, China) in a laboratory microwave oven. After inactivating the endogenous peroxidase activity with 3% H_2_O_2_/phosphate-buffered saline and blocking non-specific binding sites with 10% goat serum, sections were incubated with the primary antibodies (anti-POLR2J3, 1:200, Affinity; anti-MYH11, 1:200, Affinity) at 4 °C overnight. Then the next day, after rewarming for 1 h, sections were incubated with secondary antibodies (ZSGB-Bio, China) for 1 h at 37 °C. The slides were rinsed with PBS for three times, and then DAB was added. Three minutes later, the slides were processed with counter-stain with hematoxylin, dehydration, and mounting. The staining intensity was quantified using the Image-Pro Plus software.

### Statistical analysis

All data were analyzed by R (version 4.0.0). Comparisons between two groups were calculated using Wilcoxon test or Student t-test. A *p* value < 0.05 was considered as significant difference unless specified.

## Results

### Identification and functional analysis of 73 DEMRGs

**Figure 1:**
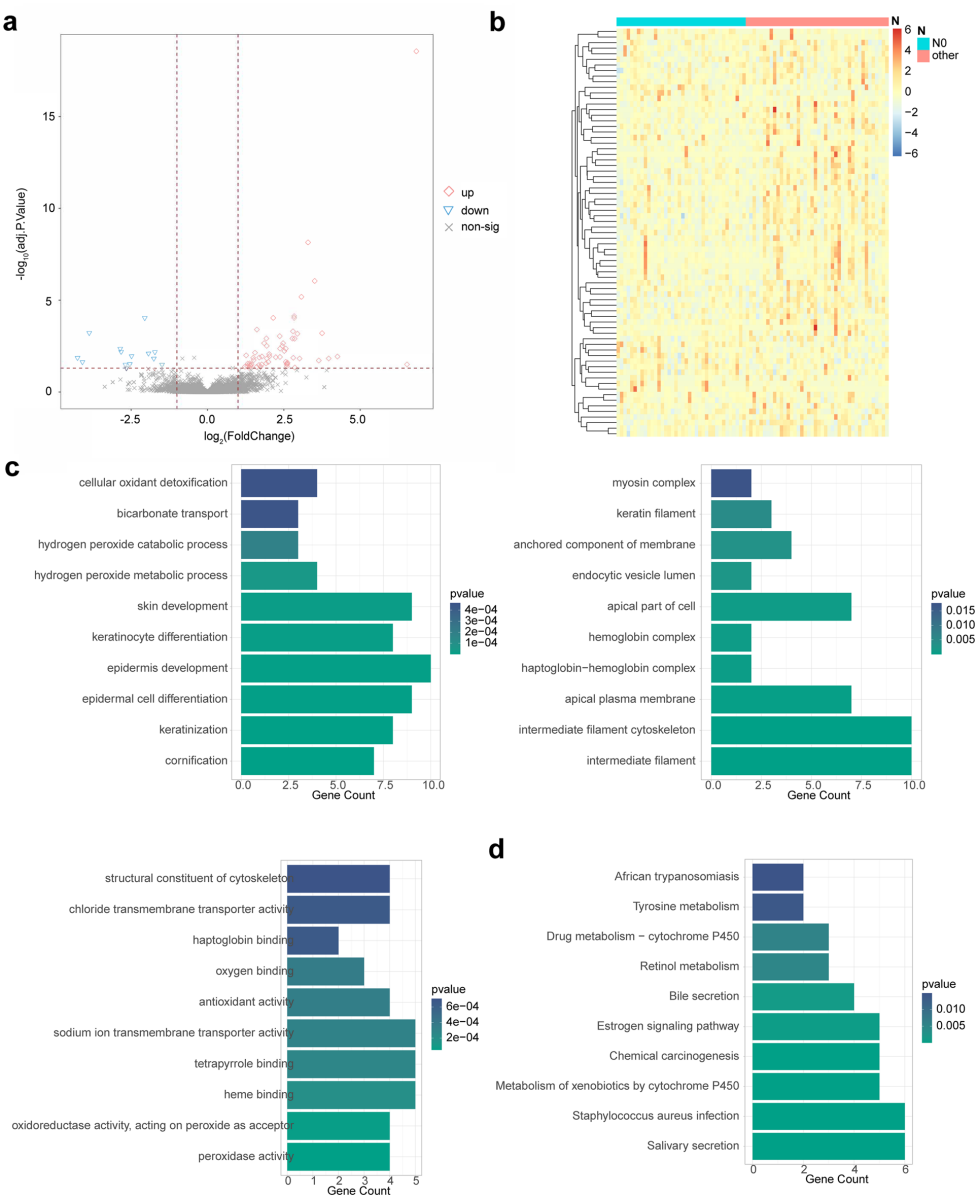
Identification of DEMRGs and functional enrichment analysis. (**a**, **b**) The volcano map (**a**) and heat map (**b**) of 73 DEMRGs. (**c**) The GO terms (including BP, CC, and MF) enrichment in DEMGs. (**d**) The KEGG pathways enriched in DEMGs. DEMGs, differentially expressed metastasis-related genes; GO, Gene ontology; BP, biological process; CC, cellular component; MF, molecular function; KEGG, Kyoto Encyclopedia of Genes and Genomes.

A total of 73 DEMRGs were identified between N0 samples and N1/N2/N3 samples of LSCC, including 59 up-regulated and 14 down-regulated genes in N0 samples relative to N1/N2/N3 samples (Fig. 1a, Supplementary Table 1). The expressions of DEMRGs were present in the heatmap (Fig. 1b). GO enrichment analysis showed that those DEMRGs were mainly enriched into BPs of skin development, keratinocyte differentiation, epidermis development, epidermal cell differentiation, keratinization and cornification, CCs of apical part of cell, apical plasma membrane, intermediate filament cytoskeleton and intermediate filament, and MFs of sodium ion transmembrane transporter activity, tetrapyrrole binding and heme binding (Fig. 1c). Meanwhile, we found that those DEMRGs were mainly involved in the KEGG pathways of salivary secretion, staphylococcus aureus infection, metabolism of xenobiotics by cytochrome P450, chemical carcinogenesis, estrogen signaling pathway and bile secretion (Fig. 1d).

### Identification of *POLR2J3* and *MYH11* as prognostic biomarkers in LSCC

**Figure 2:**
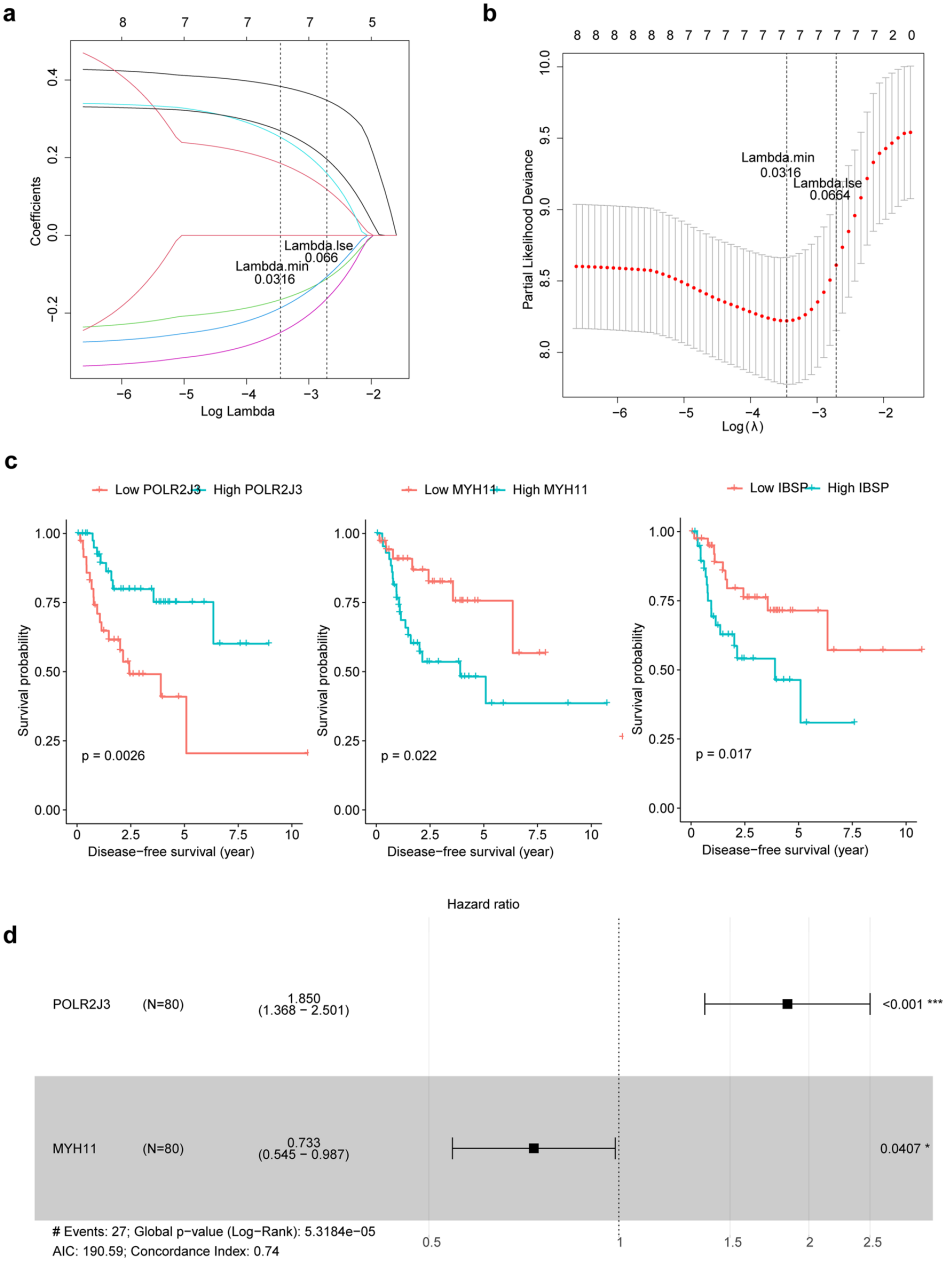
Identification of prognostic biomarkers for LSCC. (**a**) The correlation coefficient change curve in LASSO regression. (**b**) Trend of cross-validation error in LASSO regression. (**c**) DFS survival curves of *MYH11*, *IBSP* and *POLR2J3*. (**d**) The Multivariate Cox regression analysis of *POLR2J3* and *MYH11.*

Thereafter, we investigated the prognostic value of those DEMRGs. We first removed 7 DEMRGs with too low expressions. Then the remaining 66 DEMRGs were input into univariate Cox regression analysis, and *POLR2J3*, *HBB*, *MYH11*, *IBSP*, *KRT38*, *NRG2*, *VNN1* and *HBA2* were found to be significantly associated with survival (*p* < 0.05, Supplementary Table 2). Next, by LASSO regression analysis, *POLR2J3*, *HBB*, *MYH11*, *IBSP*, *KRT38*, *NRG2*, *VNN1* were selected as gene signatures (Fig. 2a,b). According to the expressions of each signature, we divided LSCC patients into low- and high-expression groups. K–M analysis showed that patients in the groups with lower expressions of *MYH11* and *IBSP* and higher expression of *POLR2J3* had better survival (Fig. 2c, Supplementary Fig. 1), suggesting that *MYH11*, *IBSP* and *POLR2J3* may play more important roles in the prognosis of LSCC patients. Finally, multivariate Cox regression analysis showed that *POLR2J3* and *MYH11* remained significantly associated with prognosis (*p* < 0.05, Fig. 2d). Thus, *POLR2J3* and *MYH11* were identified as prognostic biomarkers in LSCC and were used for the following construction of the risk model. In order to investigate the potential molecular mechanisms, we performed the GSEA enrichment analysis on *POLR2J3* and *MYH11.* The results revealed that *POLR2J3* was associated with DNA replication, oxidative phosphorylation, ribosome, selenoamino acid metabolism, spliceosome, ATP metabolic process, cellular connections, cell proliferation, WNT signalosome and so on (Supplementary Fig. 2a,b). Meanwhile, cytochrome P450, PPAR signaling pathways, immune, and MHC protein-related pathways were enriched in *MYH11* (Supplementary Fig. 2c,d)*.*

### Construction and validation of the risk score model for LSCC

**Figure 3:**
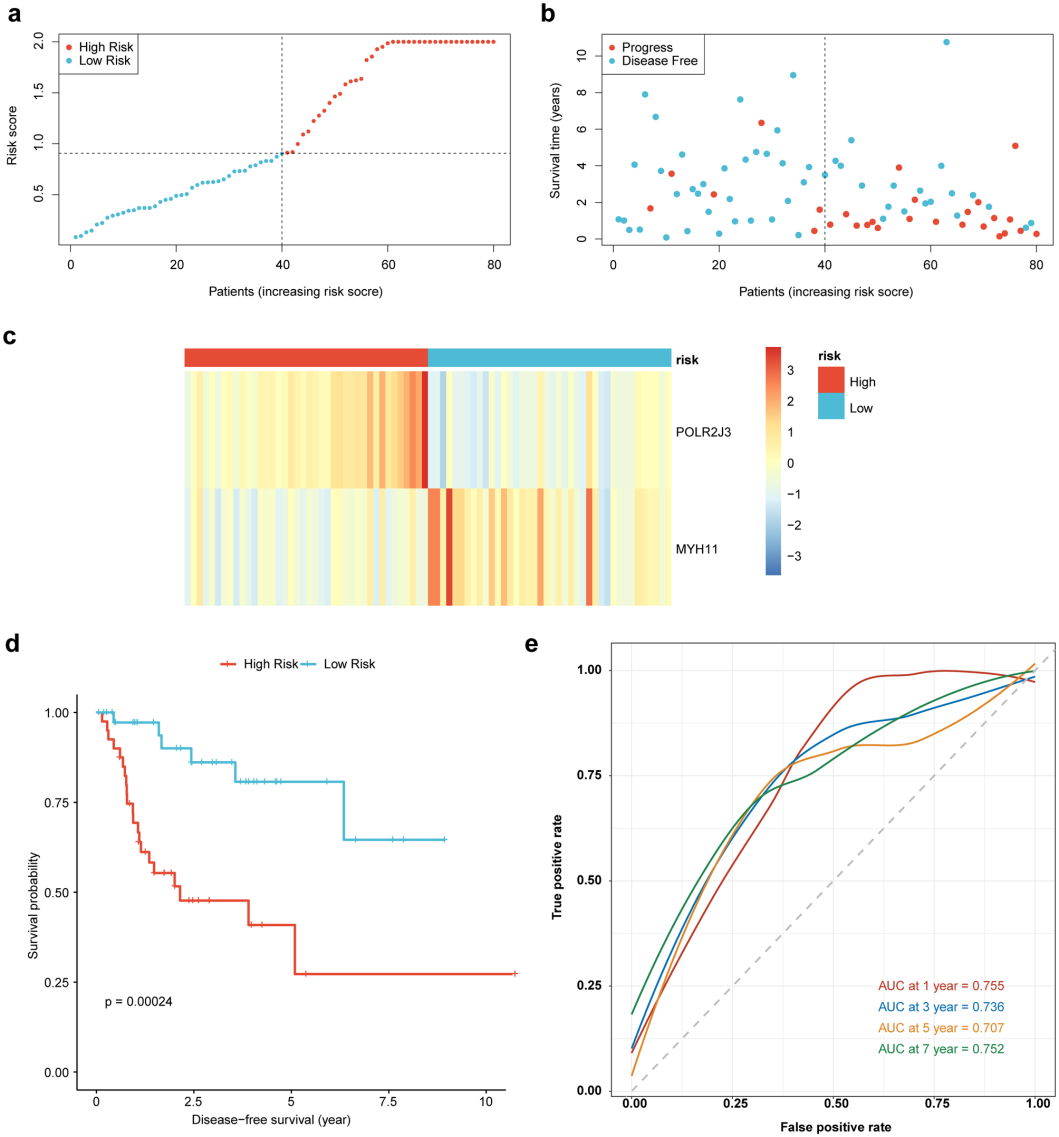
Construction of prognostic model for LSCC in the training set. (**a**) The risk curve of high- and low-risk group). (**b**) Risk curve (survival time and survival state). (**c**) The expression of prognostic biomarkers in the high- and low-risk groups. (**d**) Survival curves for high- and low-risk groups. (**e**) ROC curves of *POLR2J3* and *MYH11*.

**Figure 4:**
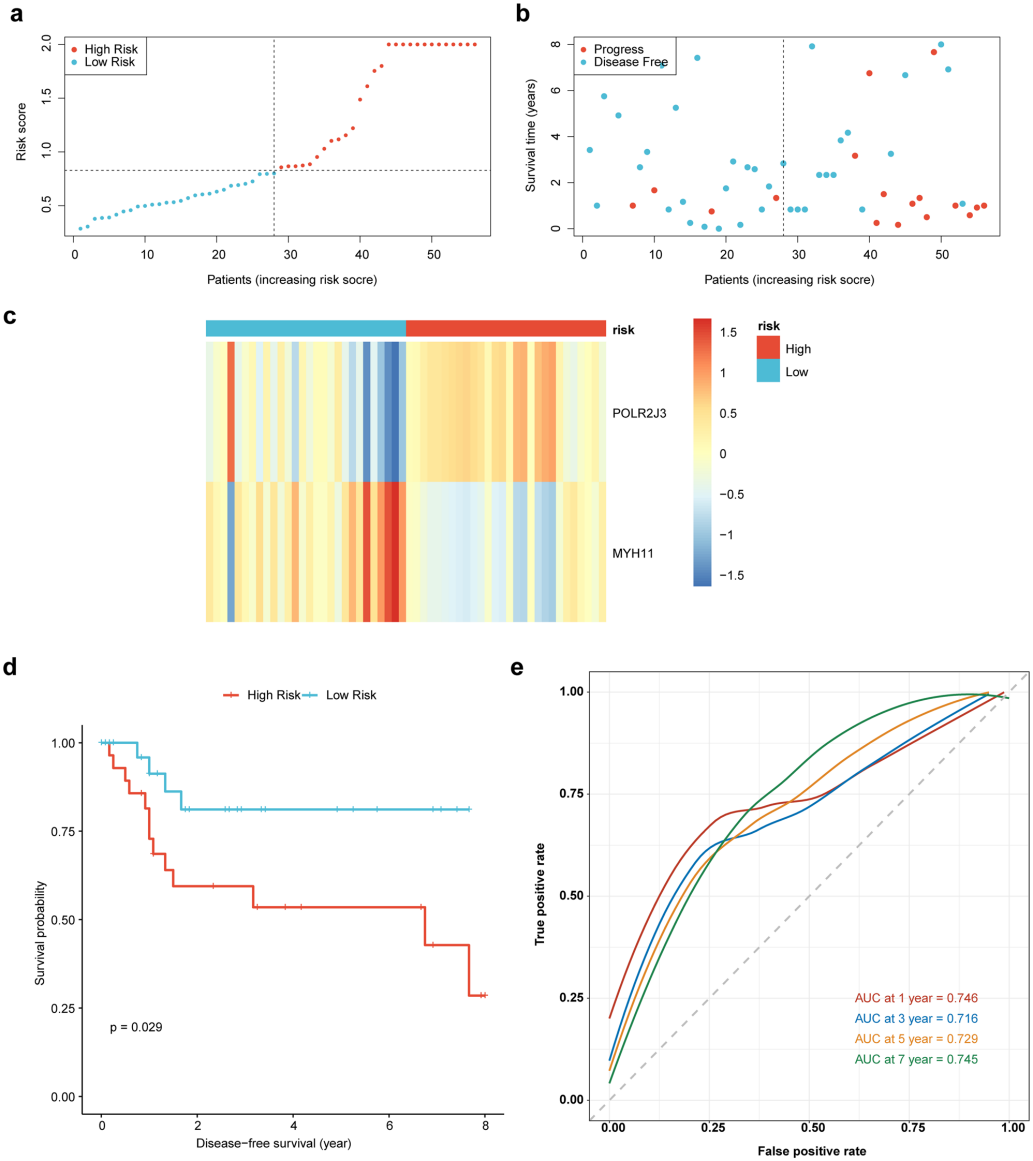
The validation of the prognostic model in the GSE25727 dataset. (**a**) Risk curve (High and low risk group) (Validation set). (**b**) Risk curve (survival time and survival state) (Validation set). (**c**) High and low risk group heat map (Validation set). (**d**) Survival curves for high- and low-risk groups (Validation set). (**e**) ROC curves (Validation set).

According to the expressions and coefficients of *POLR2J3* and *MYH11*, we calculated the risk scores of each patient. Based on the median of the risk scores, the patients in the training set were divided into high and low-risk groups (Fig. 3a), and more dead were observed in the high-risk group (Fig. 3b). We found that the expression of *POLR2J3* were much higher in the high-risk group, whereas the abundance of *MYH11* was much higher in the low-risk group (Fig. 3c). Patients in low-risk group had better survival than patients in the high-risk group (Fig. 3d). And the areas under the ROC curves for 1-, 3-, 5- and 7-year were 0.755, 0.736, 0.707 and 0.752, suggesting the good performance of the risk model in the training set (Fig. 3e). The risk model was tested in the validation set and similar results were obtained (Fig. 4a–e), indicating the reliability of the risk score model.

### Independent prognostic analysis and nomogram creation

**Figure 5:**
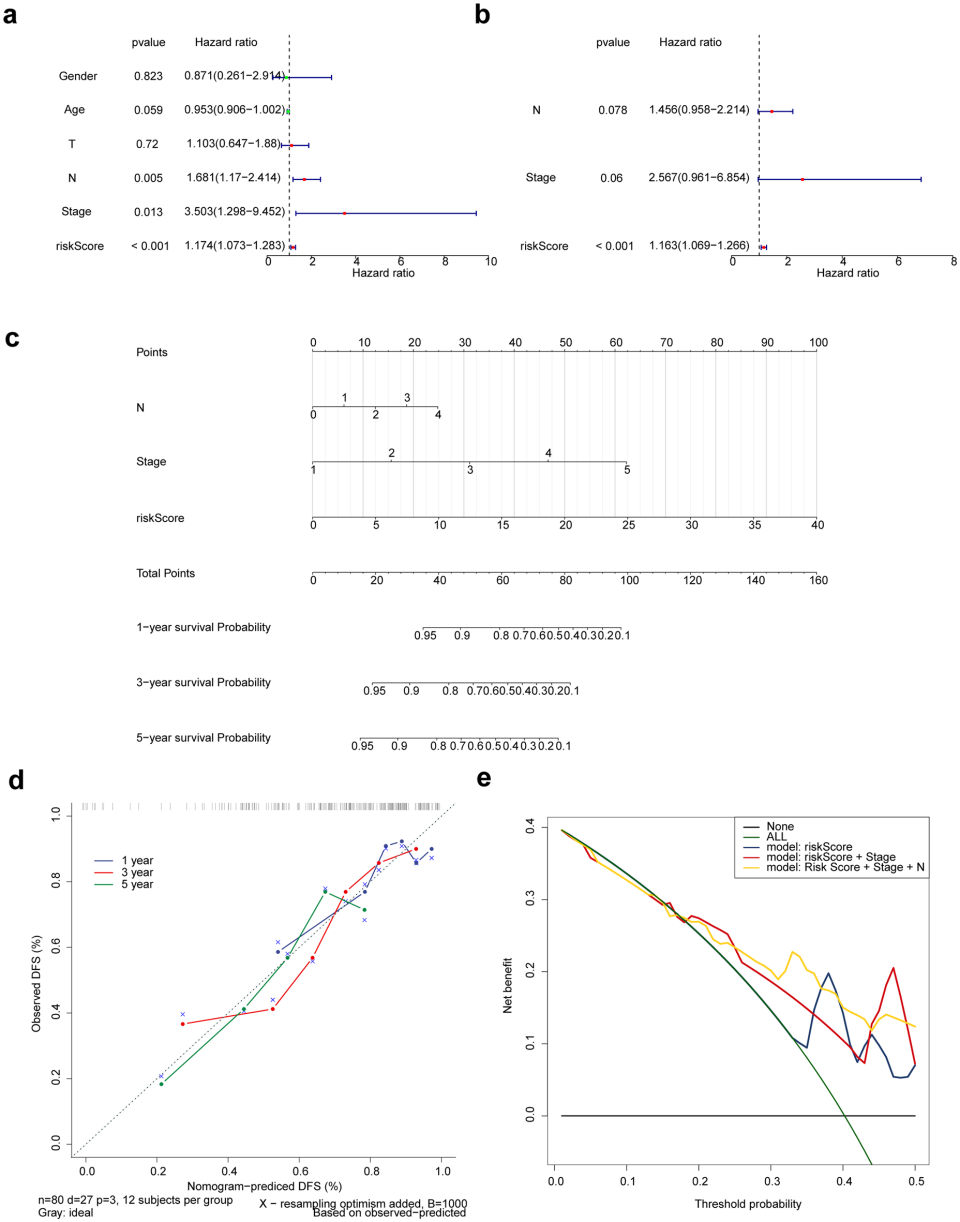
Identification of independent prognostic factors and construction of nomogram for LSCC. (**a**, **b**) The univariate (**a**) and multivariate. (**b**) Cox regression analyses of risk score and clinical factors. (**c**) Establishment of the nomogram based on independent prognostic factors. (**d**) The calibration curves of the nomogram. (**e**) The decision curve analysis (DCA) curves.

Next, we performed univariate and multivariate analyses to detect independent prognostic factors. Age, gender, T stage, N stage, tumor stage and risk score were included into univariate analysis. The univariate result showed that N stage, tumor stage and risk score were significantly associated with prognosis (Fig. 5a). N stage, tumor stage and risk score were then included into multivariate analysis, and we found that the risk score remained remarkably associated with prognosis (Fig. 5b), indicating that the risk score was an independent prognostic factor in LSCC. Thereafter, we constructed a nomogram (C-index = 0.712) to predict the 1-, 3- and 5-year survival of LSCC patients (Fig. 5c). The slope of the calibration curves for 1-, 3- and 5-year were close to 1 (Fig. 5d), indicating the high accuracy of the nomogram. In addition, the decision curves, which displayed the clinical utility of each model, showed that the nomogram had better performance in predicting the survival of LSCC patients compared to the risk model (Fig. 5e).

### Functional analysis of genes in low- and high-risk groups

**Figure 6:**
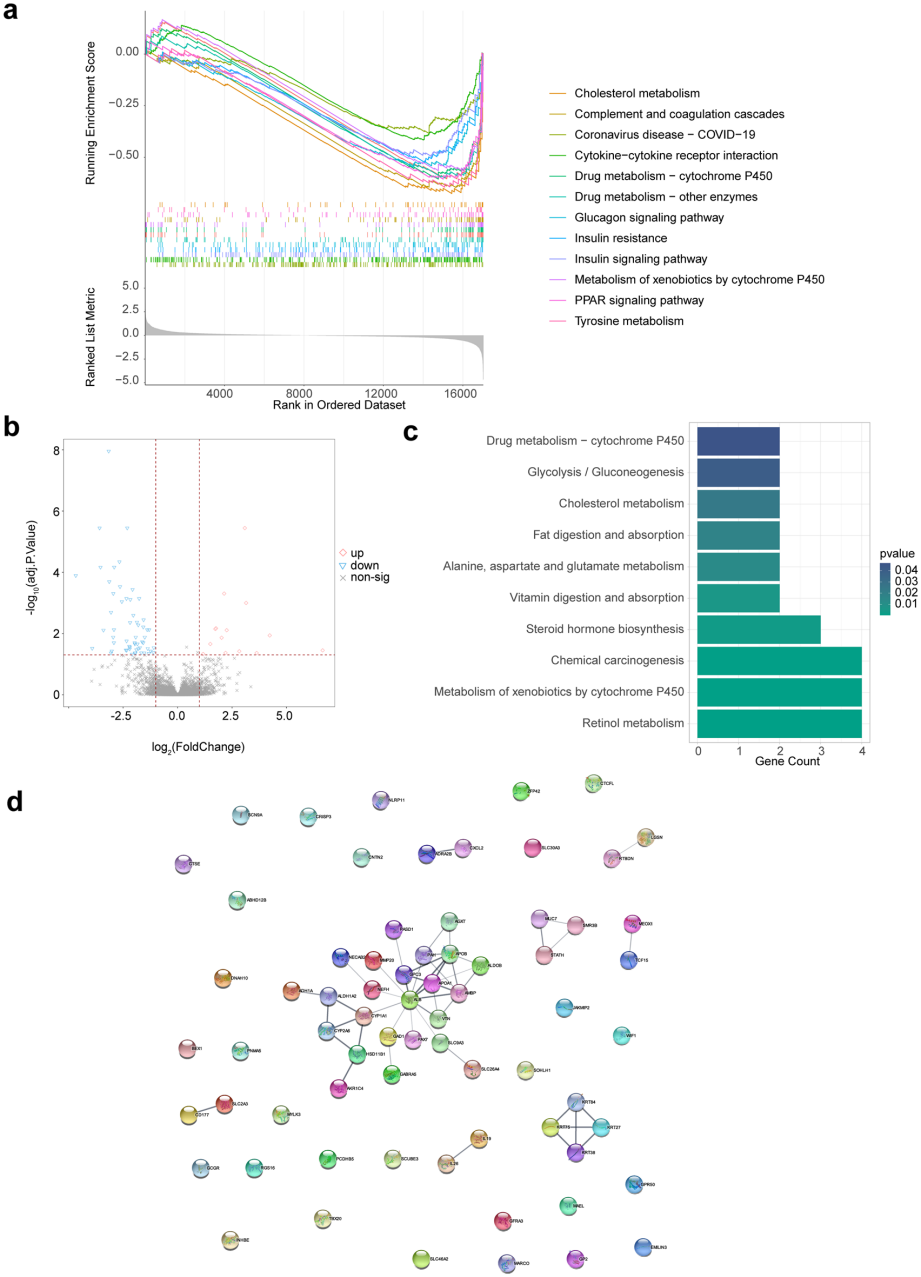
The function of genes were analyzed in high- and low-risk groups by GSEA. (**a**) Significantly active signaling pathways in the low-risk group. (**b**) 71 DEGs were identified between high- and low-risk group. (**c**) 71 DEGs were mainly enriched into various cellular metabolic processes. (**d**) Those DEGs had interactions with each other.

To better understand the underlying mechanisms of prognostic biomarkers in regulating LSCC, we first analyzed the function of genes in high- and low-risk groups by GSEA. We found that cholesterol metabolism, complement and coagulation cascades, cytokine-cytokine receptor interaction, drug metabolism-cytochrome P450 and insulin signaling pathway were significantly enriched in the low-risk group (Fig. 6a). In addition, we identified a total of 71 DEGs between high- and low-risk group (Fig. 6b), which were significantly enriched into retinol metabolism, metabolism of xenobiotics by cytochrome P450, steroid hormone biosynthesis (Fig. 6c). We speculated that prognostic biomarkers may affect the development of LSCC via those pathways. Moreover, we found that those DEGs had interactions with each other (Fig. 6d), indicating that the mechanisms involved in the development of LSCC were complex and need cooperation of numerous genes.

### The expressions of *POLR2J3* and *MYH11* in metastatic and non-metastatic LSCC tissues

**Figure 7:**
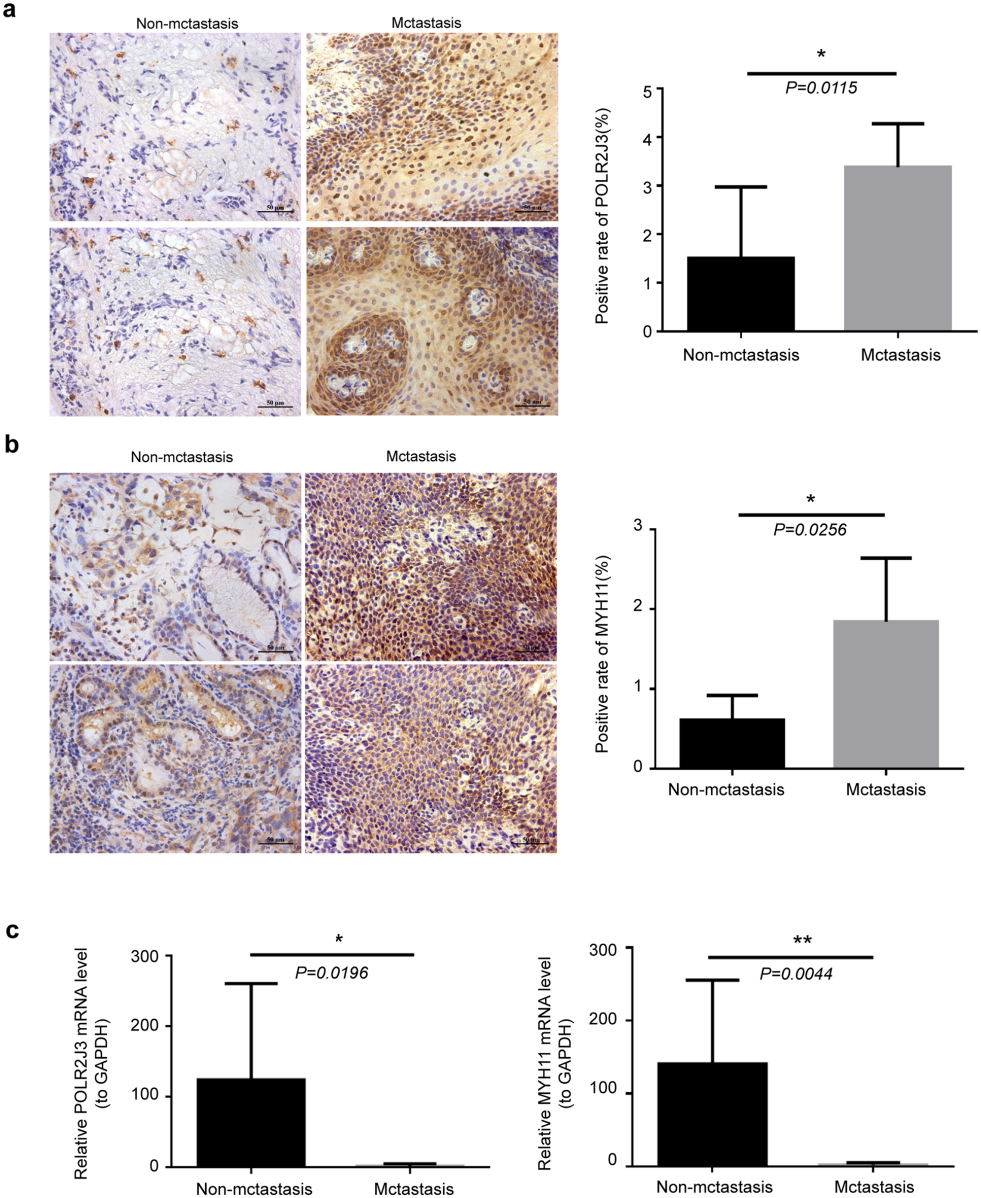
The expressions of *POLR2J3* and *MYH11* in metastatic and non-metastatic LSCC tissues. The mRNA and protein expression levels of *POLR2J3* (**a**) and *MYH11* (**b**) using IHC and RT-qPCR(**c**), respectively. *p < 0.05; **p < 0.01.

To verify the bioinformatic analysis, we detected the mRNA and protein expression levels of *POLR2J3* and *MYH11* using RT-qPCR and IHC, respectively. In accordance with our bioinformatic analysis, we found that both the mRNA and protein expression levels of *POLR2J3* and *MYH11* were significantly elevated in metastatic LSCC tissues compared with those in non-metastatic LSCC tissues (Fig. 7), further demonstrating their important role in the metastasis of LSCC.

## Discussion

Although the incidence of laryngeal cancer has been slowly decreasing over the last decade, there has been an anomalous increase in observed mortality^15^. On the one hand, due to the non-specific symptoms of laryngeal cancer and the lack of early diagnostic indicators, LSCC is not easy to detect and diagnose, and about 60% of HNSCC patients are in the advanced stage when receiving treatment, resulting in delayed treatment^16^. On the other hand, metastasis and recurrence are the main causes of poor prognosis, which reduces the overall survival rate of laryngeal cancer patients by more than 50%^4,17^. Therefore, there is an urgent need to explore new and effective prognostic indicators for laryngeal cancer. At present, the development of bioinformatics technology and gene sequencing technology has made a great contribution to the screening of molecular biomarkers and prognostic indicators.

In this study, 73 DEGs that may be involved in metastasis were identified, and two genes significantly related to the prognosis of patients with LSCC were identified by K–M curve, univariate regression, and multivariate regression: *POLR2J3* and *MYH11*. The survival rate of patients with low *MYH11* expression was high, and the survival rate of patients with high *POLR2J3* expression was low. Moreover, the mRNA expression levels of *POLR2J3* and *MYH11* in metastatic LSCC tissues were higher than those in non-metastatic LSCC tissues by qRT-PCR and IHC experiments. Compared with adjacent tissues, POLR2J3 protein and MYH11 protein were significantly overexpressed in laryngeal cancer tissues.

*POLR2J3* is the hRPB11 subunit (*POLR2J*) subtype of RNA polymerase II, and its distribution with the *POLR2J4* gene on human chromosome 7^18^. In humans, the *POLR2J* gene family has been identified, which may encode several hRPB11 proteins that are distinct mainly in the short C-terminal region. However, the function of human specific isoforms remains largely unknown^19^. Some studies have found that *POLR2J* is overexpressed in rectal tumor organoids^20^. In addition, *POLR2J* is strongly positively correlated with pentapeptide-repeat domain 1 (*PTCD1*)^21^, a mitochondrial matrix protein containing eight PPR domains^22^ that regulate mitochondrial metabolism and oxidative phosphorylation. *PTCD1* may be involved in immune function and immune cell infiltration, and may play a role in tumor progression and metastasis^21^. Cancer elicits an immune response that solid tumors can evade, in part by establishing an immunosuppressive microenvironment. The tumor microenvironment includes tissue-resident cells (such as fibroblasts, endothelial cells), innate immune cells (such as macrophages), and adaptive immune cells (such as T cells), and tumor-associated macrophages typically promote tumor growth and metastasis. They also contribute to immunosuppressive microenvironments that allow tumors to evade inherent anti-tumor immune responses and treatments. *POLR2J* and its subtype *POLR2J3* rarely appear in tumor studies, and it is even less reported in LSCC. Functional enrichment analysis revealed *POLR2J* might be involved in the genesis and development of LSCC through DNA replication, cell cycle and metabolism-related pathways.

Myosin heavy chain 11 (*MYH11*) is a smooth muscle Myosin encoded by the *MYH11* gene, which belongs to the Myosin heavy chain family^23^. Studies have shown that *MYH11* gene mutations are also related to various types of tumors, such as acute myeloid leukemia subtypes^24^, gastric cancer^25^, bladder cancer^26^, etc. *MYH11* is involved in cell adhesion, migration, and tumor inhibition, and is associated with poor prognosis^27^. The pathogenic variants in *MYH11* were reported in 2% of families with familial thoracic aortic aneurysms and dissections (FTAAD)/patent ductus arteriosus (PDA)^28^. The *MYH11* gene has a single nucleotide repeat (C8) in its coding sequence, which may be a mutation target that is unstable in cancer^29,30^. On this basis, *MYH11* mutations may increase cell migration and adhesion by altering *MYH1* function (motility and energy dysregulation) in affected cancer cells. It can lead to tumor occurrence and invasion^31^. This has been reported in the study of biomarkers of lung cancer^32^. In addition, in the study of head and neck tumors, Islam et al. believed that *MYH11* could identify high-risk and low-risk groups of head and neck tumors^33^. Su et al.^34^ used SVM-RFE to find that *MYH11* was a characteristic gene for laryngeal cancer recurrence and believed that it may be used as a prognostic factor for survival time. This coincides with the present study. According to our study, immune reponse, MHC protein, and carcinogenesis-related pathways, such as cytochrome P450 and PPAR signaling pathways were associated with *MYH11*.

We calculated the risk score and constructed the risk model, and the results of the ROC curve showed that the risk model had a good prediction effect. In addition, the results of univariate and multivariate analyses indicated that the risk score was an independent prognostic factor. At the same time, we also constructed a nomogram to predict the survival of patients with laryngeal squamous cell carcinoma at 1, 3, and 5 years. The correction curve showed that the prediction model had high prediction accuracy for 1, 3, and 5 years, which had clinical value. In addition, the decision curve results showed that the nomogram was better than the risk model in predicting the survival of patients.

At present, our results suggest that the expression of *POLR2J3* and *MYH11* genes is associated with the prognosis of laryngeal cancer patients. The survival rate of patients with low *MYH11* expression was high, and the survival rate of patients with high *POLR2J3* expression was low. Moreover, in LSCC tissue samples, the expression of these two genes was verified to be higher in metastatic cancer than in non-metastatic cancer, and higher in cancer tissues than in adjacent tissues. It is confirmed that *POLR2J3* and *MYH11* genes play a crucial role in the development, invasion and metastasis of LSCC, and may become important molecular markers and new therapeutic targets for the occurrence and prognosis of LSCC. We are the first to systematically mine metastasis-related prognostic biomarkers and construct corresponding prognostic model between metastatic and non-metastatic LSCC patients. The results of this study will provide the theoretical basis for the subsequent exploration of the molecular mechanism of the occurrence, development, invasion and metastasis of LSCC, and open a new perspective for improving the prognosis and treatment of LSCC patients. However, there are still some shortcomings in our study. Firstly, the number of non-metastatic LSCC samples and metastatic LSCC samples in this study is still insufficient, and the occurrence and development of LSCC are regulated by multiple factors and mechanisms. Then, the identification of prognostic biomarkers and the construction of the risk score model were implemented based on public database, and more clinical samples were needed to verify the database-based bioinformatics analysis. Besides, the specific mechanism of the two in the gene regulatory network of the occurrence and development of LSCC still needs to be further explored by more basic experimental data.

In conclusion, the expression of *POLR2J3* and *MYH11* genes is related to the prognosis of patients with laryngeal cancer, and they are involved in the process of the occurrence, development, invasion and metastasis of LSCC, which may become important molecular markers and new therapeutic targets for the occurrence and prognosis of LSCC.

### Supplementary Information

Supplementary Information 1.

Supplementary Information 2.

Supplementary Information 3.

## Data Availability

All data generated or analysed during this study are included in this published article.
