# Supplementary material for: Identification of a two metastasis-related prognostic signature in the process of predicting the survival of laryngeal squamous cell carcinoma
Source: Sci Rep. 2023 Aug 19;13:13513. doi: 10.1038/s41598-023-40740-2 (PMC10439939; doi:10.1038/s41598-023-40740-2)
Supplement: Supplementary file 1 — Supplementary Information 1. [file 41598_2023_40740_MOESM1_ESM.docx]

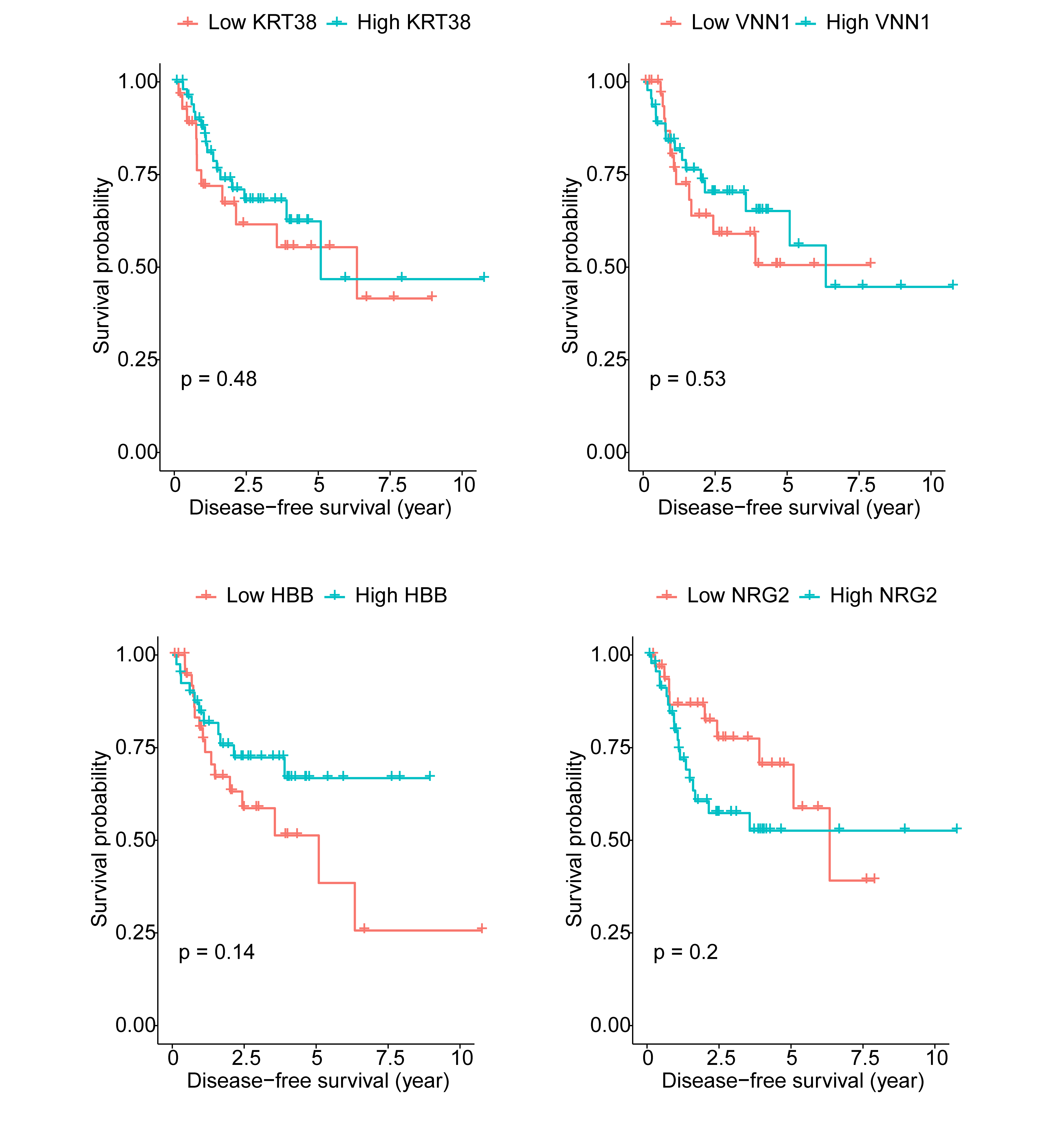


Supplementary Figure 1 Survival curves of *KRT38*, *VNN1, HBB*, and *NRG2*.


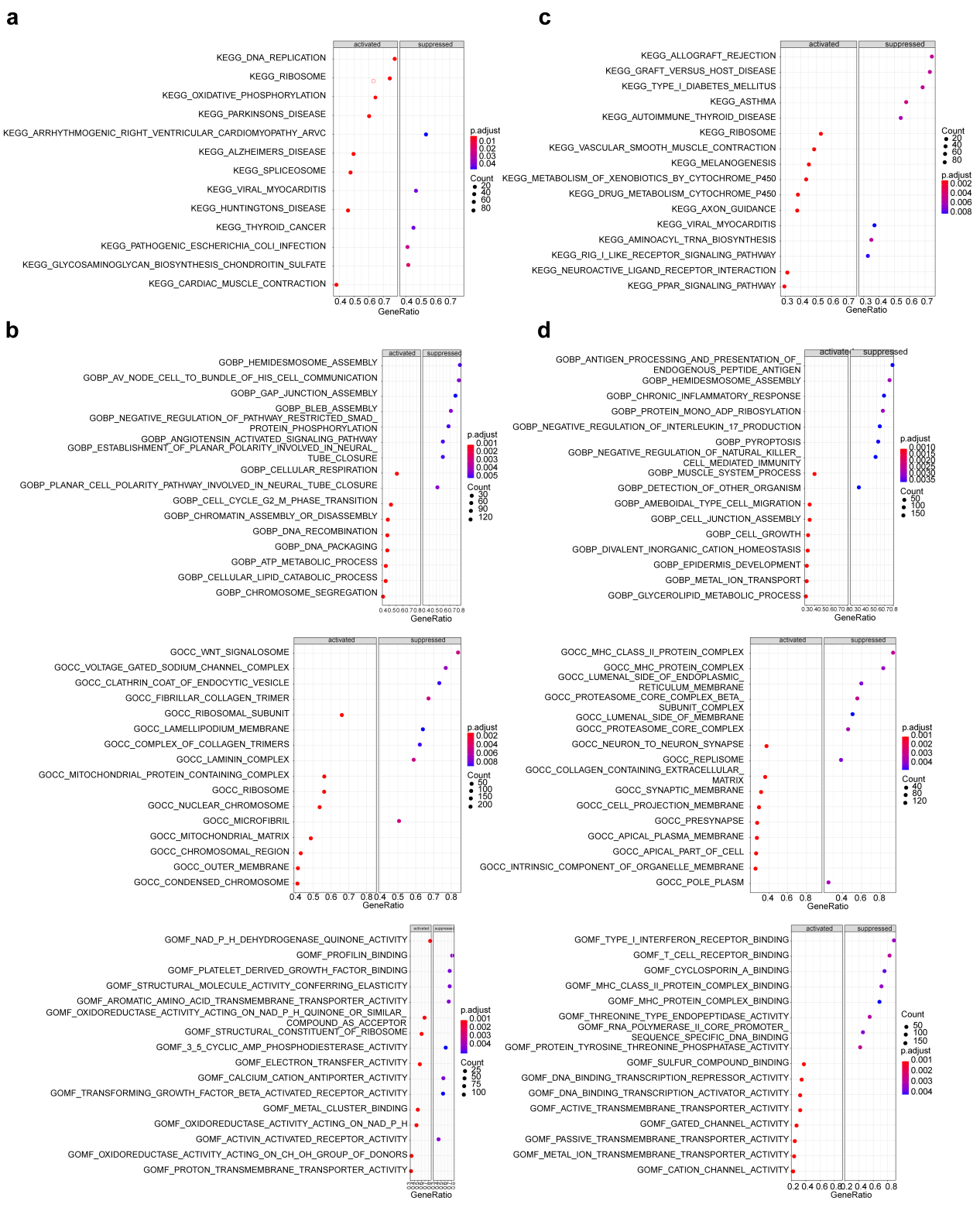


Supplementary Figure 2 Gene Set Enrichment Analysis (GSEA) for biomarkers. (a,b) The KEGG pathways (a) and GO terms (b) enriched in *POLR2J3. (c,d)* The KEGG pathways (c) and GO terms (d) enriched in *MYH11.* GO, Gene ontology; BP, biological process; CC, cellular component; MF, molecular function; KEGG, Kyoto Encyclopedia of Genes and Genomes
