# Supplementary material for: Identification of a two metastasis-related prognostic signature in the process of predicting the survival of laryngeal squamous cell carcinoma
Source: Sci Rep. 2023 Aug 19;13:13513. doi: 10.1038/s41598-023-40740-2 (PMC10439939; doi:10.1038/s41598-023-40740-2)
Supplement: Supplementary file 2 — Supplementary Information 2. [file 41598_2023_40740_MOESM2_ESM.docx]

Table S1. Identified DEMRGs in the present study.

| baseMean | log2FoldChange | lfcSE | stat | pvalue | padj |  |
| --- | --- | --- | --- | --- | --- | --- |
| PRH2 | 124.9513811 | 6.838177216 | 0.685204442 | 9.979761946 | 1.87E-23 | 2.78E-19 |
| PRR4 | 152.5341435 | 3.292322267 | 0.461345821 | 7.136343535 | 9.58E-13 | 7.14E-09 |
| KRT36 | 54.39088834 | 3.515632687 | 0.551294078 | 6.377055058 | 1.81E-10 | 8.96E-07 |
| KRT84 | 114.0977993 | 3.075216151 | 0.511167104 | 6.016068182 | 1.79E-09 | 6.65E-06 |
| KRT33B | 24.22147668 | 2.840080207 | 0.509612481 | 5.573019331 | 2.50E-08 | 7.46E-05 |
| BEST2 | 86.52507236 | 2.837895717 | 0.518291256 | 5.475484459 | 4.36E-08 | 9.24E-05 |
| NEFH | 95.32203317 | 2.156748137 | 0.392446667 | 5.495646466 | 3.89E-08 | 9.24E-05 |
| SLC22A1 | 78.44523933 | -2.050642577 | 0.376089804 | -5.45253435 | 4.97E-08 | 9.24E-05 |
| POLR2J3 | 412.1806216 | 1.61718457 | 0.313127776 | 5.164615518 | 2.41E-07 | 0.000398611 |
| KRT40 | 13.43891343 | 2.799232679 | 0.548472607 | 5.103687297 | 3.33E-07 | 0.000495984 |
| CTCFL | 21.13245753 | -3.874471924 | 0.767618837 | -5.047390369 | 4.48E-07 | 0.000606274 |
| MUC7 | 43.77344553 | 3.758696447 | 0.747916591 | 5.025555645 | 5.02E-07 | 0.00062287 |
| FABP7 | 29.27967836 | 2.944240862 | 0.589365319 | 4.99561268 | 5.86E-07 | 0.00067176 |
| KRT3 | 206.8643862 | 2.364598351 | 0.480408487 | 4.922057824 | 8.56E-07 | 0.000910831 |
| LPO | 33.5858138 | 2.780890787 | 0.573425114 | 4.84961457 | 1.24E-06 | 0.001213912 |
| HBB | 712.0592661 | 1.927118758 | 0.398240601 | 4.839081583 | 1.30E-06 | 0.001213912 |
| CEACAM7 | 699.8367765 | 2.853024231 | 0.591673346 | 4.821958349 | 1.42E-06 | 0.001245116 |
| B4GALNT2 | 213.5229935 | 2.474446659 | 0.528052196 | 4.685988768 | 2.79E-06 | 0.002183435 |
| RFPL1S | 16.49551486 | 1.855652528 | 0.395191938 | 4.695572836 | 2.66E-06 | 0.002183435 |
| CRB2 | 71.29376321 | 2.448994491 | 0.532121145 | 4.602325078 | 4.18E-06 | 0.003077696 |
| MSMB | 99.79710911 | 1.93907344 | 0.422054348 | 4.594369061 | 4.34E-06 | 0.003077696 |
| TRPM1 | 13.48058789 | 2.377641301 | 0.528935405 | 4.495144927 | 6.95E-06 | 0.004313297 |
| TGM6 | 36.70904154 | 2.563422778 | 0.569213113 | 4.503449973 | 6.69E-06 | 0.004313297 |
| ZFP42 | 47.394424 | -2.857509241 | 0.635501188 | -4.496465618 | 6.91E-06 | 0.004313297 |
| ADH1C | 99.01439938 | 2.514473123 | 0.568856304 | 4.420225466 | 9.86E-06 | 0.005872495 |
| FGF21 | 3.897120733 | -2.827763926 | 0.643496652 | -4.394372398 | 1.11E-05 | 0.006362224 |
| GPR17 | 17.72338491 | -1.720314362 | 0.393097437 | -4.376305213 | 1.21E-05 | 0.006656821 |
| MYH11 | 1621.68378 | 1.55592043 | 0.357650957 | 4.350387993 | 1.36E-05 | 0.007226801 |
| IBSP | 113.1811527 | -1.929360772 | 0.446559607 | -4.320499976 | 1.56E-05 | 0.007993161 |
| PRB3 | 24.87179327 | 2.019109428 | 0.469941623 | 4.296511161 | 1.74E-05 | 0.008611742 |
| ETNK2 | 1278.417461 | 1.265510136 | 0.297500916 | 4.253802477 | 2.10E-05 | 0.010094974 |
| CYP1A1 | 30.95996349 | -2.479651057 | 0.586888788 | -4.225078257 | 2.39E-05 | 0.01111435 |
| NLRP7 | 137.2448802 | 2.028848993 | 0.481257533 | 4.215724122 | 2.49E-05 | 0.011234188 |
| FA2H | 395.9899901 | 1.782220249 | 0.42512821 | 4.192194747 | 2.76E-05 | 0.011753259 |
| GP2 | 11.82680034 | 4.254561621 | 1.013588145 | 4.197525043 | 2.70E-05 | 0.011753259 |
| CHRM1 | 13.34747003 | 2.372997717 | 0.568375536 | 4.175052523 | 2.98E-05 | 0.012322191 |
| LDLRAD1 | 21.06226583 | 2.236449524 | 0.537421293 | 4.161445688 | 3.16E-05 | 0.012726492 |
| DLGAP2 | 11.75954772 | 1.695694442 | 0.409112002 | 4.144817152 | 3.40E-05 | 0.013325961 |
| SLC26A4 | 40.4036518 | 1.497640515 | 0.363788478 | 4.116789308 | 3.84E-05 | 0.013715332 |
| DPPA2 | 23.80817632 | -4.255317249 | 1.034052078 | -4.115186593 | 3.87E-05 | 0.013715332 |
| KRT38 | 10.64237729 | 2.809224292 | 0.68206115 | 4.118727907 | 3.81E-05 | 0.013715332 |
| STATH | 595.2879555 | 3.96819046 | 0.971231125 | 4.085732383 | 4.39E-05 | 0.014926952 |
| KEL | 37.83465103 | 1.94231121 | 0.475493827 | 4.0848295 | 4.41E-05 | 0.014926952 |
| COL2A1 | 143.1487467 | -1.756545133 | 0.431381566 | -4.07190587 | 4.66E-05 | 0.015026582 |
| DPP10 | 12.18277916 | 3.015226979 | 0.740843113 | 4.069993936 | 4.70E-05 | 0.015026582 |
| SLC1A1 | 280.8067737 | 1.513053423 | 0.376535819 | 4.018351898 | 5.86E-05 | 0.018180264 |
| KRTAP3-2 | 5.318549943 | 3.644776722 | 0.911276892 | 3.999636943 | 6.34E-05 | 0.01927791 |
| KRT20 | 10.72173454 | 2.564344548 | 0.648635279 | 3.953446 | 7.70E-05 | 0.022940623 |
| ELF5 | 293.1267826 | 1.992957498 | 0.504871504 | 3.947454914 | 7.90E-05 | 0.023060927 |
| PASD1 | 6.314682694 | -4.094336032 | 1.039733032 | -3.937872422 | 8.22E-05 | 0.023539739 |
| SCGB3A1 | 267.5672337 | 2.618476052 | 0.668324445 | 3.917971383 | 8.93E-05 | 0.025087488 |
| SLC9A2 | 290.4423692 | 1.915577712 | 0.492444775 | 3.889934078 | 0.000100271 | 0.027648926 |
| SLC9A3 | 280.9794864 | 1.381781617 | 0.355694366 | 3.884744178 | 0.000102438 | 0.027732678 |
| SCN11A | 9.351690746 | 1.318005672 | 0.340041759 | 3.876011215 | 0.000106183 | 0.028233246 |
| DCT | 24.85052828 | 2.616671176 | 0.678136226 | 3.858621728 | 0.000114028 | 0.029787385 |
| TUBA3E | 3.811844544 | -2.542999381 | 0.660256523 | -3.851532387 | 0.000117381 | 0.029906652 |
| GSTA1 | 460.6069536 | 2.590235433 | 0.672927394 | 3.849204915 | 0.000118502 | 0.029906652 |
| NRG2 | 31.36183789 | 1.441942087 | 0.375491213 | 3.840148678 | 0.00012296 | 0.030514529 |
| SCUBE3 | 244.1249883 | 1.774248999 | 0.463849186 | 3.825055756 | 0.000130742 | 0.031399286 |
| SMR3B | 17.74423474 | 6.531637417 | 1.706921942 | 3.826558939 | 0.000129947 | 0.031399286 |
| UGT2B4 | 1.660119689 | -2.687630588 | 0.705559799 | -3.809217294 | 0.000139407 | 0.032434004 |
| HTR1D | 57.68425417 | -1.488253031 | 0.390535172 | -3.810804088 | 0.000138516 | 0.032434004 |
| VNN1 | 256.5771807 | 1.390930132 | 0.36683383 | 3.791717172 | 0.000149609 | 0.033856671 |
| PRRG3 | 50.88425046 | 1.686950852 | 0.444993777 | 3.790953806 | 0.00015007 | 0.033856671 |
| CGNL1 | 606.1057345 | 1.417378725 | 0.377253547 | 3.757098468 | 0.000171895 | 0.038201711 |
| SCGB1A1 | 58.39214426 | 2.602645841 | 0.695480041 | 3.742229378 | 0.000182395 | 0.039360293 |
| FCGR3B | 103.7593251 | 1.299798477 | 0.347054328 | 3.745230567 | 0.000180228 | 0.039360293 |
| KLHDC8A | 132.6906403 | 1.739002771 | 0.4673399 | 3.721066339 | 0.000198383 | 0.041618746 |
| PRH1 | 12.52563615 | 1.225706706 | 0.32940422 | 3.720980578 | 0.000198451 | 0.041618746 |
| IL17REL | 13.30320515 | 1.609020263 | 0.433974429 | 3.707638414 | 0.000209201 | 0.043263945 |
| HOXC12 | 8.81637374 | 2.859335718 | 0.775569055 | 3.686758387 | 0.000227129 | 0.046328055 |
| SULT2A1 | 1.216719057 | -2.656630816 | 0.725047059 | -3.664080532 | 0.000248229 | 0.049415088 |
| HBA2 | 189.6687014 | 1.367070701 | 0.373342708 | 3.661704576 | 0.000250543 | 0.049415088 |
