# Supplementary material for: Identification of a two metastasis-related prognostic signature in the process of predicting the survival of laryngeal squamous cell carcinoma
Source: Sci Rep. 2023 Aug 19;13:13513. doi: 10.1038/s41598-023-40740-2 (PMC10439939; doi:10.1038/s41598-023-40740-2)
Supplement: Supplementary file 3 — Supplementary Information 3. [file 41598_2023_40740_MOESM3_ESM.docx]

Table S2. By Cox regression analysis, POLR2J3, HBB, MYH11, IBSP, KRT38, NRG2, VNN1 and HBA2 were found to be significantly associated with survival**(*P***＜0.05**)**.

|  | characteristics | Hazard.Ration | CI95 | pvalue | HR(95%CI) |  |
| --- | --- | --- | --- | --- | --- | --- |
| 1 | PRH2 | 0.922 | 0.811-1.048 | 0.215 | 0.9220(0.811-1.048) |  |
| 2 | PRR4 | 0.8904 | 0.740-1.072 | 0.22 | 0.8904(0.740-1.072) |  |
| 3 | KRT36 | 1.0966 | 0.997-1.206 | 0.058 | 1.0966(0.997-1.206) |  |
| 4 | KRT84 | 1.0653 | 0.949-1.196 | 0.284 | 1.0653(0.949-1.196) |  |
| 5 | KRT33B | 1.0375 | 0.879-1.224 | 0.663 | 1.0375(0.879-1.224) |  |
| 6 | BEST2 | 1.1273 | 0.968-1.313 | 0.123 | 1.1273(0.968-1.313) |  |
| 7 | NEFH | 1.1528 | 0.983-1.351 | 0.079 | 1.1528(0.983-1.351) |  |
| 8 | SLC22A1 | 0.8361 | 0.657-1.064 | 0.146 | 0.8361(0.657-1.064) |  |
| 9 | POLR2J3 | 1.8239 | 1.350-2.464 | < 0.001 | 1.8239(1.350-2.464) |  |
| 10 | KRT40 | 0.9916 | 0.823-1.195 | 0.93 | 0.9916(0.823-1.195) |  |
| 11 | CTCFL | 1.0278 | 0.927-1.140 | 0.603 | 1.0278(0.927-1.140) |  |
| 12 | MUC7 | 0.9246 | 0.807-1.059 | 0.257 | 0.9246(0.807-1.059) |  |
| 13 | FABP7 | 0.8623 | 0.709-1.049 | 0.139 | 0.8623(0.709-1.049) |  |
| 14 | KRT3 | 0.9714 | 0.849-1.111 | 0.672 | 0.9714(0.849-1.111) |  |
| 15 | LPO | 0.9599 | 0.827-1.114 | 0.59 | 0.9599(0.827-1.114) |  |
| 16 | HBB | 1.2142 | 1.022-1.443 | 0.027 | 1.2142(1.022-1.443) |  |
| 17 | CEACAM7 | 1.0791 | 0.964-1.208 | 0.187 | 1.0791(0.964-1.208) |  |
| 18 | B4GALNT2 | 0.9606 | 0.834-1.107 | 0.578 | 0.9606(0.834-1.107) |  |
| 19 | RFPL1S | 1.2046 | 0.980-1.480 | 0.077 | 1.2046(0.980-1.480) |  |
| 20 | CRB2 | 1.063 | 0.901-1.255 | 0.47 | 1.0630(0.901-1.255) |  |
| 21 | MSMB | 1.0767 | 0.933-1.243 | 0.313 | 1.0767(0.933-1.243) |  |
| 22 | TRPM1 | 1.0428 | 0.858-1.268 | 0.674 | 1.0428(0.858-1.268) |  |
| 23 | TGM6 | 1.1057 | 0.949-1.289 | 0.198 | 1.1057(0.949-1.289) |  |
| 24 | ZFP42 | 0.9461 | 0.820-1.092 | 0.448 | 0.9461(0.820-1.092) |  |
| 25 | ADH1C | 0.9947 | 0.879-1.126 | 0.933 | 0.9947(0.879-1.126) |  |
| 26 | FGF21 | 0.9383 | 0.732-1.203 | 0.615 | 0.9383(0.732-1.203) |  |
| 27 | GPR17 | 0.8267 | 0.648-1.055 | 0.127 | 0.8267(0.648-1.055) |  |
| 28 | MYH11 | 0.7588 | 0.578-0.996 | 0.047 | 0.7588(0.578-0.996) |  |
| 29 | IBSP | 0.8057 | 0.680-0.954 | 0.012 | 0.8057(0.680-0.954) |  |
| 30 | PRB3 | 0.8439 | 0.698-1.020 | 0.08 | 0.8439(0.698-1.020) |  |
| 31 | ETNK2 | 1.0589 | 0.836-1.342 | 0.636 | 1.0589(0.836-1.342) |  |
| 32 | CYP1A1 | 0.9704 | 0.842-1.119 | 0.678 | 0.9704(0.842-1.119) |  |
| 33 | NLRP7 | 1.0691 | 0.928-1.232 | 0.355 | 1.0691(0.928-1.232) |  |
| 34 | FA2H | 1.0886 | 0.896-1.323 | 0.393 | 1.0886(0.896-1.323) |  |
| 35 | CHRM1 | 0.9789 | 0.815-1.175 | 0.819 | 0.9789(0.815-1.175) |  |
| 36 | LDLRAD1 | 0.9961 | 0.838-1.184 | 0.964 | 0.9961(0.838-1.184) |  |
| 37 | DLGAP2 | 1.1033 | 0.878-1.387 | 0.4 | 1.1033(0.878-1.387) |  |
| 38 | SLC26A4 | 1.0265 | 0.786-1.341 | 0.848 | 1.0265(0.786-1.341) |  |
| 39 | KRT38 | 1.2516 | 1.068-1.467 | 0.006 | 1.2516(1.068-1.467) |  |
| 40 | STATH | 0.9528 | 0.864-1.051 | 0.333 | 0.9528(0.864-1.051) |  |
| 41 | KEL | 1.114 | 0.940-1.321 | 0.214 | 1.1140(0.940-1.321) |  |
| 42 | COL2A1 | 0.8594 | 0.718-1.029 | 0.1 | 0.8594(0.718-1.029) |  |
| 43 | DPP10 | 1.0287 | 0.887-1.193 | 0.707 | 1.0287(0.887-1.193) |  |
| 44 | SLC1A1 | 1.0669 | 0.860-1.323 | 0.555 | 1.0669(0.860-1.323) |  |
| 45 | KRT20 | 1.1024 | 0.930-1.307 | 0.261 | 1.1024(0.930-1.307) |  |
| 46 | ELF5 | 1.033 | 0.893-1.194 | 0.661 | 1.0330(0.893-1.194) |  |
| 47 | SCGB3A1 | 0.9294 | 0.821-1.052 | 0.247 | 0.9294(0.821-1.052) |  |
| 48 | SLC9A2 | 0.97 | 0.843-1.116 | 0.67 | 0.9700(0.843-1.116) |  |
| 49 | SLC9A3 | 1.1714 | 0.924-1.485 | 0.191 | 1.1714(0.924-1.485) |  |
| 50 | SCN11A | 0.979 | 0.725-1.322 | 0.89 | 0.9790(0.725-1.322) |  |
| 51 | DCT | 0.9997 | 0.873-1.145 | 0.996 | 0.9997(0.873-1.145) |  |
| 52 | TUBA3E | 0.9216 | 0.751-1.131 | 0.435 | 0.9216(0.751-1.131) |  |
| 53 | GSTA1 | 1.0401 | 0.941-1.149 | 0.441 | 1.0401(0.941-1.149) |  |
| 54 | NRG2 | 0.7506 | 0.568-0.992 | 0.044 | 0.7506(0.568-0.992) |  |
| 55 | SCUBE3 | 0.9457 | 0.804-1.112 | 0.5 | 0.9457(0.804-1.112) |  |
| 56 | HTR1D | 0.888 | 0.718-1.098 | 0.273 | 0.8880(0.718-1.098) |  |
| 57 | VNN1 | 1.2936 | 1.046-1.599 | 0.017 | 1.2936(1.046-1.599) |  |
| 58 | PRRG3 | 0.8682 | 0.712-1.058 | 0.162 | 0.8682(0.712-1.058) |  |
| 59 | CGNL1 | 0.9076 | 0.727-1.134 | 0.393 | 0.9076(0.727-1.134) |  |
| 60 | SCGB1A1 | 1.0485 | 0.929-1.183 | 0.443 | 1.0485(0.929-1.183) |  |
| 61 | FCGR3B | 1.2212 | 0.975-1.529 | 0.081 | 1.2212(0.975-1.529) |  |
| 62 | KLHDC8A | 0.8955 | 0.730-1.099 | 0.291 | 0.8955(0.730-1.099) |  |
| 63 | PRH1 | 0.844 | 0.673-1.058 | 0.142 | 0.8440(0.673-1.058) |  |
| 64 | IL17REL | 1.0644 | 0.886-1.278 | 0.504 | 1.0644(0.886-1.278) |  |
| 65 | HOXC12 | 0.8628 | 0.682-1.091 | 0.218 | 0.8628(0.682-1.091) |  |
| 66 | HBA2 | 1.2178 | 1.003-1.479 | 0.047 | 1.2178(1.003-1.479) |  |
